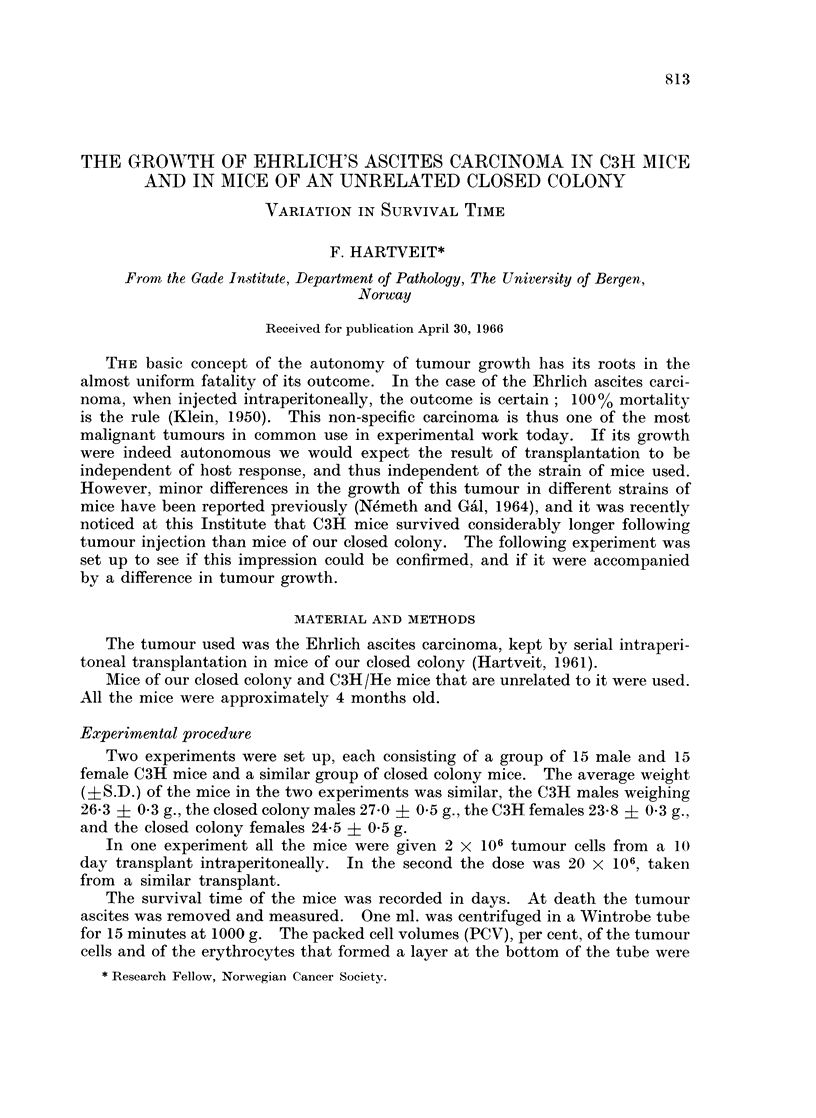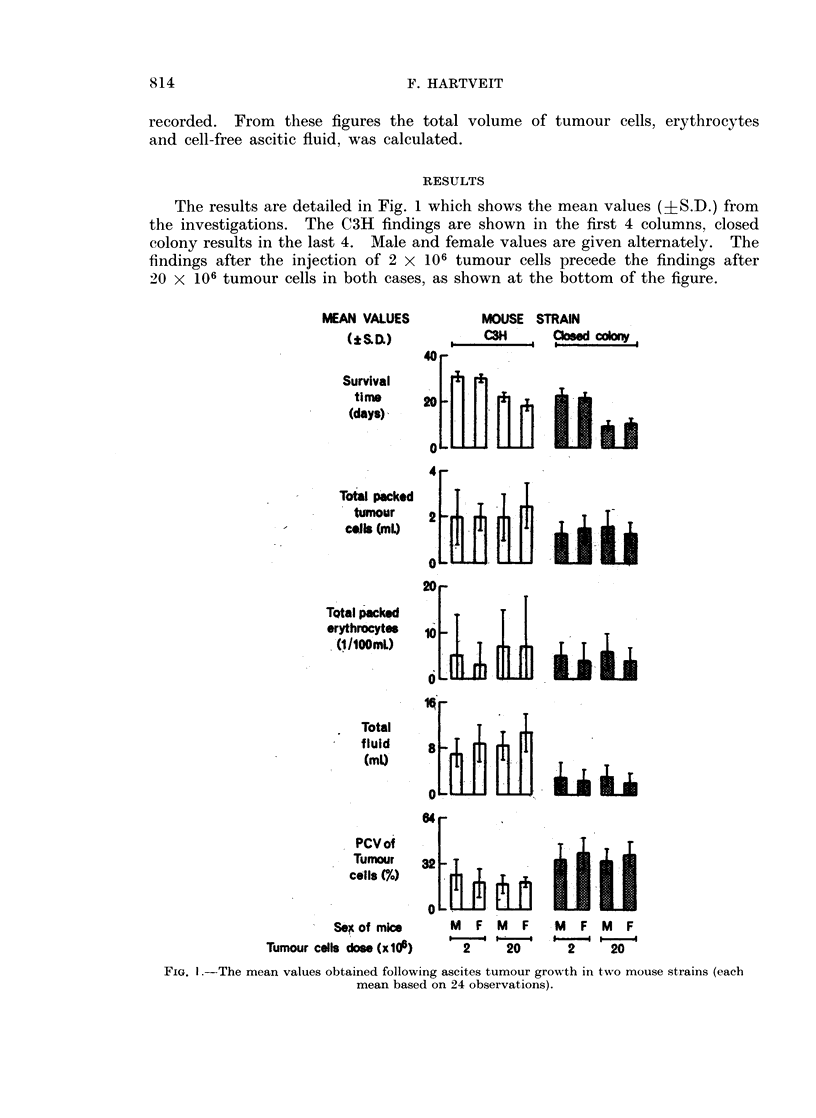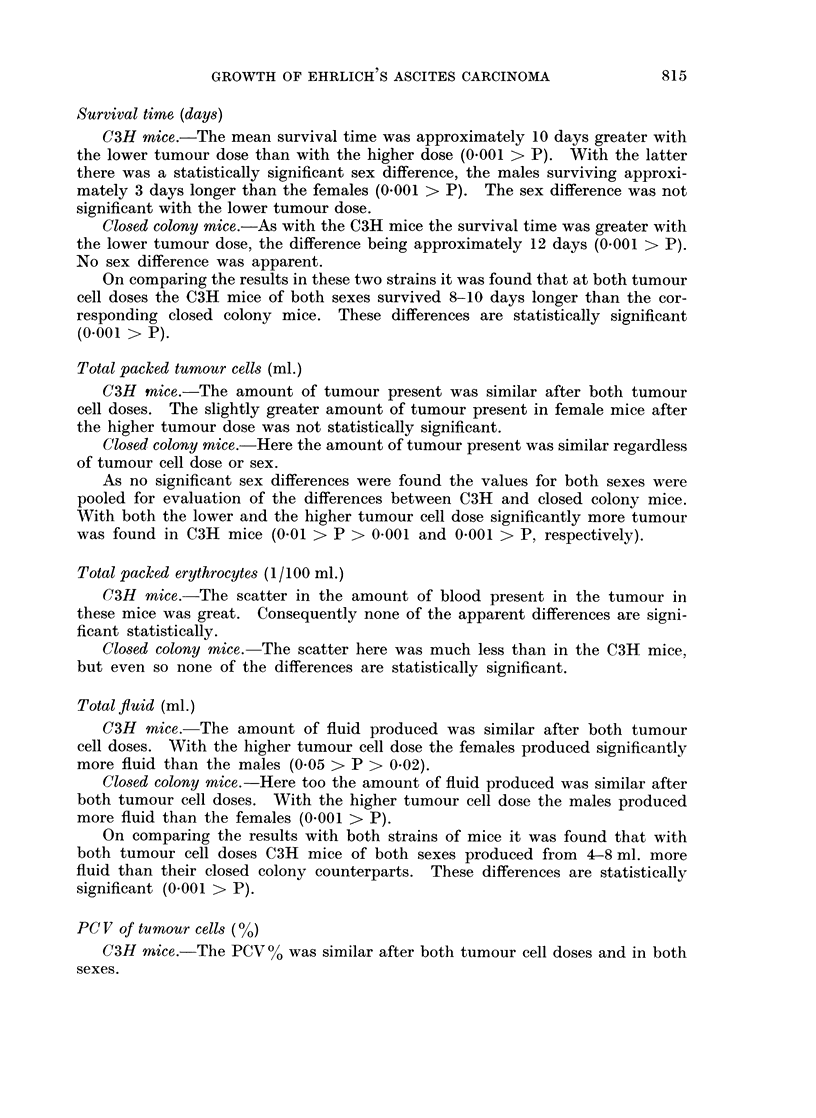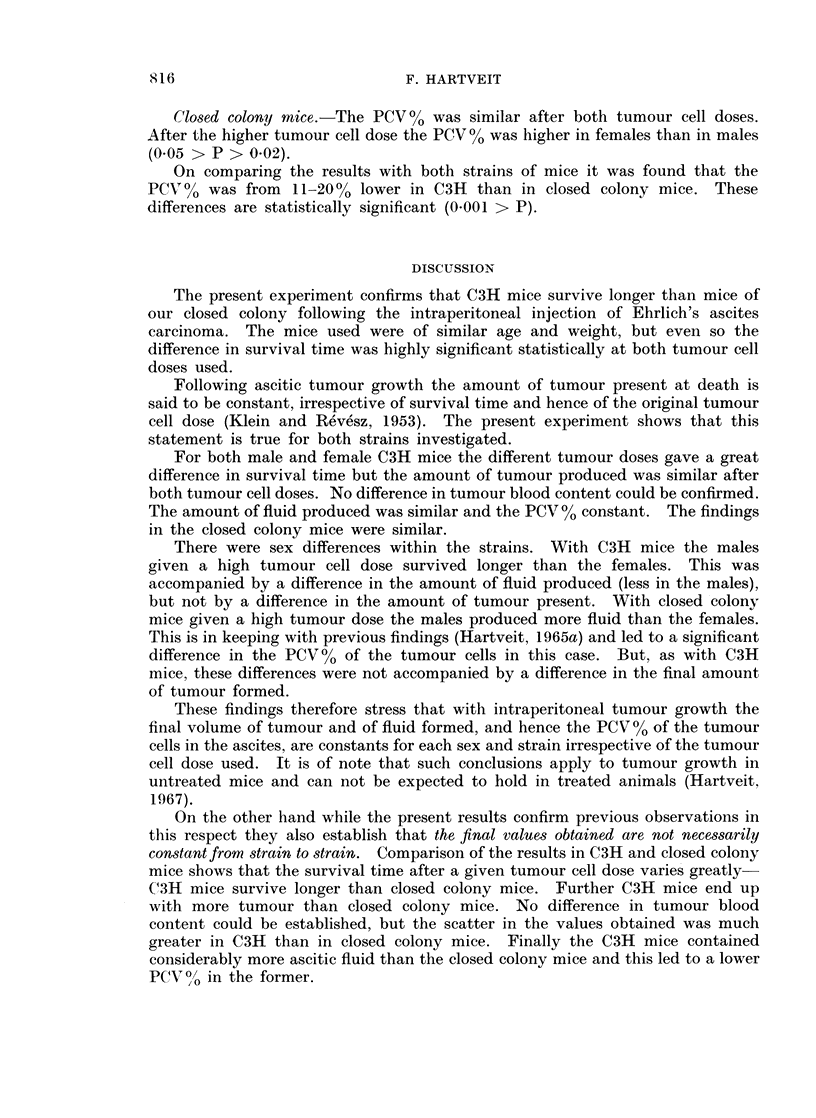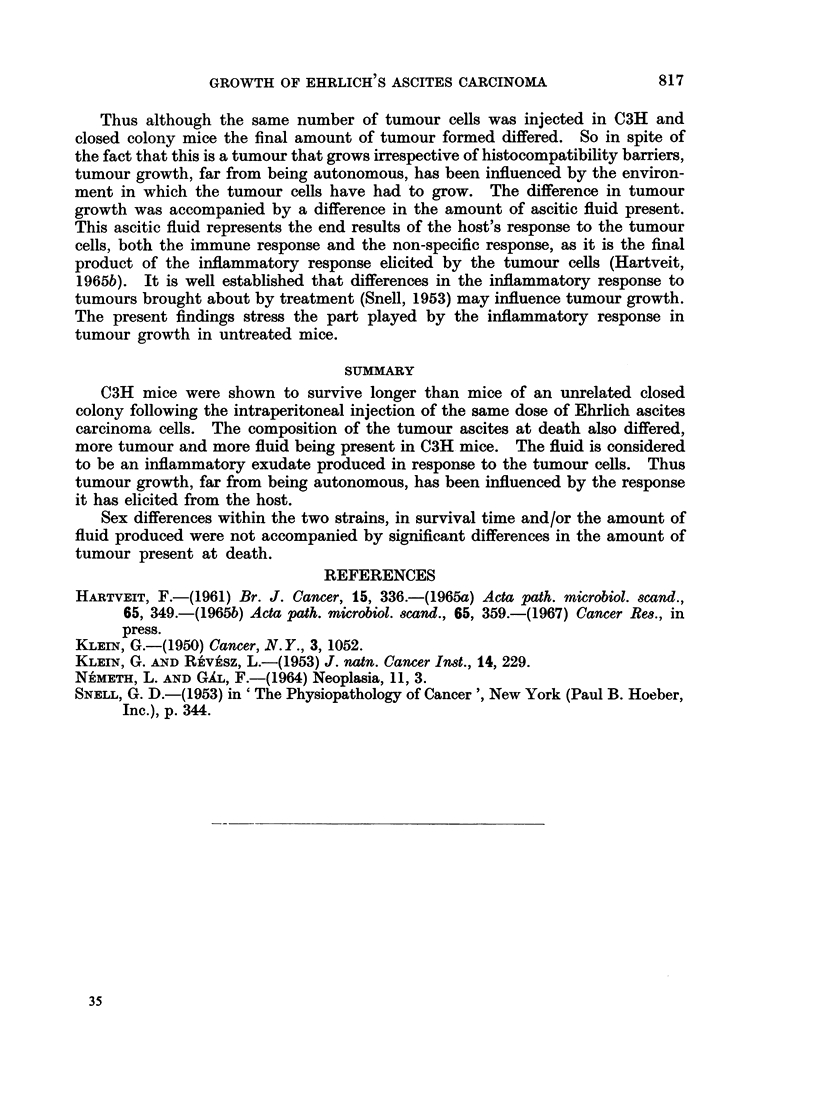# The growth of Ehrlich's ascites carcinoma in C3H mice and in mice of an unrelated closed colony. Variation in survival time.

**DOI:** 10.1038/bjc.1966.92

**Published:** 1966-12

**Authors:** F. Hartveit


### THE GROWTH OF EHRLICH'S ASCITES CARCINOMA IN C3H MICE AND IN MICE OF AN UNRELATED CLOSED COLONY

### Variation in Survival Time

F. HARTVEIT*

*From the Gade Institute, Department of Pathology, The University of Bergen, Norway*

Received for publication April 30, 1966

The basic concept of the autonomy of tumour growth has its roots in the almost uniform fatality of its outcome. In the case of the Ehrlich ascites carcinoma, when injected intraperitoneally, the outcome is certain ; 100% mortality is the rule (Klein, 1950). This non-specific carcinoma is thus one of the most malignant tumours in common use in experimental work today. If its growth were indeed autonomous we would expect the result of transplantation to be independent of host response, and thus independent of the strain of mice used. However, minor differences in the growth of this tumour in different strains of mice have been reported previously (Németh and Gál, 1964), and it was recently noticed at this Institute that C3H mice survived considerably longer following tumour injection than mice of our closed colony. The following experiment was set up to see if this impression could be confirmed, and if it were accompanied by a difference in tumour growth.

### MATERIAL AND METHODS

The tumour used was the Ehrlich ascites carcinoma, kept by serial intraperitoneal transplantation in mice of our closed colony (Hartveit, 1961).

Mice of our closed colony and C3H/He mice that are unrelated to it were used. All the mice were approximately 4 months old.

### *Experimental procedure*

Two experiments were set up, each consisting of a group of 15 male and 15 female C3H mice and a similar group of closed colony mice. The average weight (±S.D.) of the mice in the two experiments was similar, the C3H males weighing 26·3 ± 0·3 g., the closed colony males 27·0 ± 0·5 g., the C3H females 23·8 ± 0·3 g., and the closed colony females 24·5 ± 0·5 g.

In one experiment all the mice were given 2 × $10^6$ tumour cells from a 10 day transplant intraperitoneally. In the second the dose was 20 × $10^6$, taken from a similar transplant.

The survival time of the mice was recorded in days. At death the tumour ascites was removed and measured. One ml. was centrifuged in a Wintrobe tube for 15 minutes at 1000 g. The packed cell volumes (PCV), per cent, of the tumour cells and of the erythrocytes that formed a layer at the bottom of the tube were

* Research Fellow, Norwegian Cancer Society.

recorded. From these figures the total volume of tumour cells, erythrocytes and cell-free ascitic fluid, was calculated.

RESULTS

The results are detailed in Fig. 1 which shows the mean values ($\pm$S.D.) from the investigations. The C3H findings are shown in the first 4 columns, closed colony results in the last 4. Male and female values are given alternately. The findings after the injection of $2 \times 10^6$ tumour cells precede the findings after $20 \times 10^6$ tumour cells in both cases, as shown at the bottom of the figure.

FIG. 1.—The mean values obtained following ascites tumour growth in two mouse strains (each mean based on 24 observations).

*Survival time* (*days*)

*C3H mice.*—The mean survival time was approximately 10 days greater with the lower tumour dose than with the higher dose ($0{\cdot}001 > P$). With the latter there was a statistically significant sex difference, the males surviving approximately 3 days longer than the females ($0{\cdot}001 > P$). The sex difference was not significant with the lower tumour dose.

*Closed colony mice.*—As with the C3H mice the survival time was greater with the lower tumour dose, the difference being approximately 12 days ($0{\cdot}001 > P$). No sex difference was apparent.

On comparing the results in these two strains it was found that at both tumour cell doses the C3H mice of both sexes survived 8–10 days longer than the corresponding closed colony mice. These differences are statistically significant ($0{\cdot}001 > P$).

*Total packed tumour cells* (ml.)

*C3H mice.*—The amount of tumour present was similar after both tumour cell doses. The slightly greater amount of tumour present in female mice after the higher tumour dose was not statistically significant.

*Closed colony mice.*—Here the amount of tumour present was similar regardless of tumour cell dose or sex.

As no significant sex differences were found the values for both sexes were pooled for evaluation of the differences between C3H and closed colony mice. With both the lower and the higher tumour cell dose significantly more tumour was found in C3H mice ($0{\cdot}01 > P > 0{\cdot}001$ and $0{\cdot}001 > P$, respectively).

*Total packed erythrocytes* (1/100 ml.)

*C3H mice.*—The scatter in the amount of blood present in the tumour in these mice was great. Consequently none of the apparent differences are significant statistically.

*Closed colony mice.*—The scatter here was much less than in the C3H mice, but even so none of the differences are statistically significant.

*Total fluid* (ml.)

*C3H mice.*—The amount of fluid produced was similar after both tumour cell doses. With the higher tumour cell dose the females produced significantly more fluid than the males ($0{\cdot}05 > P > 0{\cdot}02$).

*Closed colony mice.*—Here too the amount of fluid produced was similar after both tumour cell doses. With the higher tumour cell dose the males produced more fluid than the females ($0{\cdot}001 > P$).

On comparing the results with both strains of mice it was found that with both tumour cell doses C3H mice of both sexes produced from 4–8 ml. more fluid than their closed colony counterparts. These differences are statistically significant ($0{\cdot}001 > P$).

*PCV of tumour cells* (%)

*C3H mice.*—The PCV % was similar after both tumour cell doses and in both sexes.

*Closed colony mice.*—The PCV% was similar after both tumour cell doses. After the higher tumour cell dose the PCV% was higher in females than in males ($0{\cdot}05 > P > 0{\cdot}02$).

On comparing the results with both strains of mice it was found that the PCV% was from 11–20% lower in C3H than in closed colony mice. These differences are statistically significant ($0{\cdot}001 > P$).

### DISCUSSION

The present experiment confirms that C3H mice survive longer than mice of our closed colony following the intraperitoneal injection of Ehrlich's ascites carcinoma. The mice used were of similar age and weight, but even so the difference in survival time was highly significant statistically at both tumour cell doses used.

Following ascitic tumour growth the amount of tumour present at death is said to be constant, irrespective of survival time and hence of the original tumour cell dose (Klein and Révész, 1953). The present experiment shows that this statement is true for both strains investigated.

For both male and female C3H mice the different tumour doses gave a great difference in survival time but the amount of tumour produced was similar after both tumour cell doses. No difference in tumour blood content could be confirmed. The amount of fluid produced was similar and the PCV% constant. The findings in the closed colony mice were similar.

There were sex differences within the strains. With C3H mice the males given a high tumour cell dose survived longer than the females. This was accompanied by a difference in the amount of fluid produced (less in the males), but not by a difference in the amount of tumour present. With closed colony mice given a high tumour dose the males produced more fluid than the females. This is in keeping with previous findings (Hartveit, 1965*a*) and led to a significant difference in the PCV% of the tumour cells in this case. But, as with C3H mice, these differences were not accompanied by a difference in the final amount of tumour formed.

These findings therefore stress that with intraperitoneal tumour growth the final volume of tumour and of fluid formed, and hence the PCV% of the tumour cells in the ascites, are constants for each sex and strain irrespective of the tumour cell dose used. It is of note that such conclusions apply to tumour growth in untreated mice and can not be expected to hold in treated animals (Hartveit, 1967).

On the other hand while the present results confirm previous observations in this respect they also establish that *the final values obtained are not necessarily constant from strain to strain.* Comparison of the results in C3H and closed colony mice shows that the survival time after a given tumour cell dose varies greatly—C3H mice survive longer than closed colony mice. Further C3H mice end up with more tumour than closed colony mice. No difference in tumour blood content could be established, but the scatter in the values obtained was much greater in C3H than in closed colony mice. Finally the C3H mice contained considerably more ascitic fluid than the closed colony mice and this led to a lower PCV% in the former.

Thus although the same number of tumour cells was injected in C3H and closed colony mice the final amount of tumour formed differed. So in spite of the fact that this is a tumour that grows irrespective of histocompatibility barriers, tumour growth, far from being autonomous, has been influenced by the environment in which the tumour cells have had to grow. The difference in tumour growth was accompanied by a difference in the amount of ascitic fluid present. This ascitic fluid represents the end results of the host's response to the tumour cells, both the immune response and the non-specific response, as it is the final product of the inflammatory response elicited by the tumour cells (Hartveit, 1965*b*). It is well established that differences in the inflammatory response to tumours brought about by treatment (Snell, 1953) may influence tumour growth. The present findings stress the part played by the inflammatory response in tumour growth in untreated mice.

### SUMMARY

C3H mice were shown to survive longer than mice of an unrelated closed colony following the intraperitoneal injection of the same dose of Ehrlich ascites carcinoma cells. The composition of the tumour ascites at death also differed, more tumour and more fluid being present in C3H mice. The fluid is considered to be an inflammatory exudate produced in response to the tumour cells. Thus tumour growth, far from being autonomous, has been influenced by the response it has elicited from the host.

Sex differences within the two strains, in survival time and/or the amount of fluid produced were not accompanied by significant differences in the amount of tumour present at death.

### REFERENCES

HARTVEIT, F.—(1961) *Br. J. Cancer*, **15**, 336.—(1965*a*) *Acta path. microbiol. scand.*, **65**, 349.—(1965*b*) *Acta path. microbiol. scand.*, **65**, 359.—(1967) *Cancer Res.*, in press.

KLEIN, G.—(1950) *Cancer, N.Y.*, **3**, 1052.

KLEIN, G. AND RÉVÉSZ, L.—(1953) *J. natn. Cancer Inst.*, **14**, 229.

NÉMETH, L. AND GÁL, F.—(1964) Neoplasia, 11, 3.

SNELL, G. D.—(1953) in ' The Physiopathology of Cancer ', New York (Paul B. Hoeber, Inc.), p. 344.